# The classification of eating disorders in China: A categorical model or a dimensional model

**DOI:** 10.1002/eat.23069

**Published:** 2019-03-18

**Authors:** Yuchen Zheng, Qing Kang, Jiabin Huang, Wenhui Jiang, Qiang Liu, Han Chen, Qing Fan, Zhen Wang, Zeping Xiao, Jue Chen

**Affiliations:** ^1^ Shanghai Mental Health Center Shanghai Jiaotong University School of Medicine Shanghai China

**Keywords:** anorexia nervosa, bulimia nervosa, classification, eating disorder, taxometric analysis

## Abstract

**Objective:**

According to the ICD‐10 and DSM‐5, eating disorders (EDs) are classified using a categorical model that assumes the subtypes are qualitatively different from one another. However, it is still intensely debated that a dimensional model is more suitable. The aim of this study is to examine whether EDs have a categorical or dimensional latent structure using a sample of Chinese ED patients.

**Method:**

The sample included 322 patients, diagnosed with an ED from 2010 to 2017 in the Shanghai Mental Health Center, and comparison participants (*N* = 850), recruited from undergraduate students in one university in Shanghai. Participants were evaluated with the Eating Disorder Inventory‐2 (EDI‐2) questionnaire and another questionnaire developed by the researchers. Three taxometric procedures (MAXimum EIGenvalue [MAXEIG], latent‐mode factor analysis [L‐Mode], and Mean Above Minus Below A Cut [MAMBAC]) were applied, respectively, to analyze the patients' clinical symptoms data.

**Results:**

Patients were divided into three groups according to their clinical diagnosis. The plots of the three taxometric analysis procedures supported the categorical construct in anorexia nervosa, binge‐eating/purging group, and bulimia nervosa group. The Comparison Curve Fit Indices of the MAXEIG, L‐Mode, and MAMBAC procedures were 0.694, 0.709, 0.704 in the AN‐BP group and 0.727, 0.67, 0.62 in the BN group, respectively, which also support the categorical construct.

**Discussion:**

The results support two distinct classes of ED subtypes among Chinese sample. Further work on applying hybrid model in analysis has been discussed.

## 1 INTRODUCTION

According to the International Classification of Diseases and Related Health Problems (ICD‐10) and the Diagnostic and Statistical Manual of Mental Disorders (DSM‐5), the two main guides for clinical diagnoses, subtypes of eating disorders (EDs) are discrete entities that are qualitatively different from normality (American Psychiatric Association, 2013; World Health Organization, 2013). From this perspective, individuals with and without an ED are distinguished using a categorical model, which assumes that the EDs can be identified by a single cutoff (Craddock & Owen, 2007) rather than by continuously varying dimensions (Krueger, Watson, & Barlow, 2005). However, the imperfection of current classification model of ED, for example, the majority of ED patients present “mixed” presentations that neither closely resemble the previous DSM‐IV or newly revised DSM‐5 diagnoses (Fairburn & Cooper, 2011), has indicated that more revisions based on empirical studies are still in need. As a result, despite the wide application of the categorical model in current diagnostic criteria, it is still intensely debated among psychopathology researchers that a dimensional model that characterizes EDs as extreme expressions of continuously distributed traits may be a better choice (Wildes & Marcus, 2013).

Many methods have been applied to address this issue by searching for homogenous subgroups of EDs in large samples (Mclachlan & Basford, 1988), including latent class analysis (e.g., Lazarsfeld & Henry, 1968), which identifies the latent structure with the categorical variables, latent profile analysis (LPA) (Mclachlan & Peel, 2000), which uses the continuous data to recover hidden groups, and growth mixture modeling (Muthén, Sayer, & Collins, 2001; Nagin & Tremblay, 2001), which identifies latent classes with variables collected during multiple longitudinal time points. These methods assume the existence of latent categories among the sample and the results would converge on a best‐fitting solution with multiple groups, even when the groups are not indeed discrete. There are also some other methods assuming latent dimensions of the data set, such as factor analysis (FA), which evaluates the factors that underlie the observed variables as being correlated or not (e.g., Williamson et al., 2002). Because the results of FA vary according to the factor solution of theoretical hypothesis, the common factors supported by different studies would be considered as having strong evidence. However, these methods could not answer whether the latent structure of the sample is dimensional or discontinuous (Williamson, Gleaves, & Stewart, 2005).

To answer the question whether two putative groups do truly differ categorically, taxometric analysis (TA) is an ideal choice. It is one of the most frequently used methods (Ruscio, Walters, Marcus, & Kaczetow, 2010) and appears to have greater scientific validity and clinical utility when compared with alternative methods (Wonderlich, Joiner Jr., Keel, Williamson, & Crosby, 2007). TA is a set of statistical procedures that can be used to identify whether the variables can distinguish groups of individuals by searching for abrupt changes in the structure of data (Beauchaine, 2007). The latent subgroups inside are taxons, and the rest are complements.

To date, there are many researchers focusing on the latent structure of EDs using TA. More attention has been paid to the potentially taxonic distinction of bulimia nervosa (BN), the results of three studies (Gleaves, Lowe, Green, Cororve, & Williams, 2001; Gleaves, Lowe, Snow, Green, & Murphy‐Eberenz, 2000; Williamson et al., 2002) have been consistently indicated that BN represents a categorically distinct class from normality. However, Tylka and Subich (2003) suggested that BN was dimensional rather than taxonic among a large sample of college women. Another evidence‐supportive distinct taxon was binge‐eating disorder (BED) (Williamson et al., 2002), separated from people with normal weight and obese non‐binge‐eating. Williamson et al. found that anorexia nervosa restricting subtype (AN‐R) was on the same dimension with normality while it was qualitatively different from anorexia nervosa binge‐eating/ purging subtype (AN‐BP). Considering the result of some latent class analysis researches that BN and AN‐BP were placed in one class (e.g., Wade, Bergin, Martin, Gillespie, & Fairburn, 2006), the results of taxometric studies (Gleaves et al., 2001; Williamson et al., 2002) indicated that the AN‐BP might have more similarity with BN than with the AN‐R subtype. Another taxonic structure was found between BED and affective or anxiety disorder, indicating the boundary of BED with psychiatric comorbidity (Hilbert, Pike, Wilfley, Fairburn, Dohm, & Streigel‐Moore, 2011). There are some other studies which supported a dimensional structure of EDs, and most of these studies were implemented among community samples (Holm‐Denoma, Richey, & Joiner Jr., 2010; Tylka & Subich, 2003). Besides, Olatunji et al. have measured the latent structure of EDs among a large clinical sample, supporting a dimensional structure among AN‐R, AN‐BP, and BN (Olatunji et al., 2012).

Some of the results among the studies mentioned above are inconsistent. Possible reasons for the inconsistency are differences in samples, the variables used, and the analytical methods employed (Gordon, Holm‐Denoma, Smith, Fink, & Joiner Jr., 2007; Wade et al., 2006; Williamson et al., 2005). For example, some studies have used clinical patient samples (Williamson et al., 2002), and others have used university student samples (Tylka & Subich, 2003), which might lead to contradictory conclusions. The diversity of indicators in different studies might also cause the disagreement of results in previous studies by influencing the result of taxonicity. Some researchers have found that when indicators of behavioral symptoms were used in TA, the construct turned to be categorical (Gleaves et al., 2000; Williamson et al., 2002), whereas the nonbehavioral indicators would lead the construct be dimensional (Tylka & Subich, 2003).

There are some other limitations of previous studies. For example, all of these studies have accepted body mass index (BMI) to describe the situation of thinness and analyzed the data of adolescents and adults together. According to the WHO, however, it is recommended to evaluate the weight of adolescents with BMI‐for‐age (Aladawi et al., 2013). This suggestion is also adopted in the latest guidelines (Aladawi et al (2013); American Psychiatric Association, 2013).

Till now, there are relatively few empirical studies focusing on EDs in Asian samples, especially by using TA. As a culture related disease, there are differences between eastern and western ED patients in their clinical characteristics. Fat phobia was one of the debates. Since non‐fat‐phobic AN subtype was first reported in a study investigating ED subtypes in Hong Kong (Lee, 1991), a series of studies about non‐western ED patients have been conducted, and evidence on the occurrence of non‐fat‐phobic AN has also been found in other Asian samples (Becker, Thomas, & Pike, 2009). Therefore, it is still unknown whether the results of latent classes from Western countries could be replicated in Chinese ED sample.

One recent study on ED patients in Hong Kong focused on the TA of Chinese ED patients (Thomas et al., 2015). In this study, patients were separated into different latent groups by LPA, with indicator variables including fat‐phobic opinion and other clinical data, and TA was applied to determine whether latent classes were qualitatively or quantitatively distinct. The findings of the taxometric procedure supported two distinct classes of low weight EDs. However, there are other limitations in the study that render the results inconsistent. The evaluation of fat‐phobic opinion in this study might be limited. The difference between ideal weight and current weight (i.e.*,* a patient whose ideal weight is lower than the current weight is considered to fear fat) may not be as an accurate indicator as the researchers proposed. For example, a patient with extremely low body weight may prefer an ideal weight higher than his/her current weight, yet still much lower than the normal standard. Meanwhile, the sample size in specific groups fell below the recommended minimum sample size of 300 for TA.

In order to solve the limitation mentioned above, z‐score of BMI/BMI‐for‐age were used to adult/adolescent separately, to measure the severity of thinness in this study. Eight subscales in Eating Disorder Inventory‐2 (EDI‐2; Garner, 1991) were collected to reflect multiple aspects of EDs.

Therefore, the purposes of this study are to analyze the latent structure of the clinical characteristics of Chinese patients with EDs, to discuss whether the best model fit is a dimensional or categorical one, and to provide empirical evidence for ED classification in future diagnostic guides and research studies in China.

## 2 METHOD

### 2.1 Sample

The participants were recruited consecutively from the ED outpatients and inpatients from the Department of Clinical Psychology in Shanghai Mental Health Center between May 2010 and December 2017. All patients were interviewed and assessed by two senior psychiatrists based on the Diagnostic and Statistical Manual of Mental Disorders, fourth edition (DSM‐IV‐TR, American Psychiatry Association, 2000) and met the diagnostic criteria of AN restricting type (*n* = 115), AN binge‐eating/purging type (*n* = 98), or BN (*n* = 109).

Those eligible for inclusion were (a) patients who met the diagnostic criteria of DSM‐IV‐TR and were diagnosed with one ED subtype by two senior psychiatrists; (b) not below 13 years of age; and (c) patients having the ability to understand and complete questionnaires. Patients who had mental retardation or a history of diabetes mellitus, hypertension, cardiopathy, liver disease, renal failure, hypothyroidism, or any other organic or neurological diseases were excluded.

All patients recruited were seeking treatment at the Shanghai Mental Health Center for the first time and were in an episode at enrollment. The total sample size of patients enrolled was 322, with 314 females and 8 males (Table 1). The demographic data of the participants are displayed in Table 2. The sample ranged in age from 11 to 40, with 19 as the median. The education level distribution of the patients was as follows: elementary school, 9 (2.8%); junior high, 61 (18.9%); high school, 99 (30.7%); undergraduate, 131 (40.7%); and postgraduate, 18 (5.6%). Their ages ranged the illness duration was from 1 to 192 months, with 18 as the median. The mean and *SD* of BMI (range, 10.85–26.62 kg/m^2^) were 16.84 and 3.19 kg/m^2^, respectively. According to the diagnostic criteria in the DSM‐IV‐TR, there were 115 (35.7%) participants with AN (restrictive type), 98 (30.4%) participants with AN (binge‐eating/purging type), and 109 (33.9%) participants with BN.

**Table 1:** Demographic characteristics of 322 eating disorder patients in Shanghai Mental Health Center

	Values	Number of patients (*n* = 322)
BMI; mean (*SD*)	16.84 (3.19)	
Age of treatment; median (minimum, maximum)	19 (11, 40)	
Age at onset of illness; median (minimum, maximum)	16 (10, 38)	
The classification of diagnoses (based on DSM‐IV) (*n*, %)		
Anorexia nervosa (restricting type)		115 (35.7%)
Anorexia nervosa (binge‐eating/purging type)		98 (30.4%)
Bulimia nervosa		109 (33.9%)
Education level (*n*, %)		
Elementary school		9 (2.8%)
Junior high		61 (18.9%)
High school		99 (30.7%)
Undergraduate		131 (40.7%)
Postgraduate		18 (5.6%)

**Table 2:** Descriptive data for different indicators included in the taxometrics analysis

	Sample size	Taxon base rate	Nuisance covariance taxon, complement	Correlation (total sample)
AN‐BP vs. HC	945	0.101	0.28, 0.27	0.372
BN vs. HC	960	0.114	0.27, 0.27	0.348

Each subtype of ED was separated and combined with nonclinical comparison sample, and the TA was applied to the mixed sample. The comparison participants (*N* = 850) were recruited from undergraduate students in one university in Shanghai. Consent forms explaining the purpose and procedures of the study were read and signed by all participants. All participants were of Han Chinese origin.

The study protocol was approved by the *Ethics Committee of the Shanghai Mental Health Center*, and informed consent forms were signed by the participants and the parents of those who were below 18 years of age.

### 2.2 Measurements

All measurements were administered prior to enrollment.

1. *Professional diagnosis*: The inpatients and outpatients were diagnosed with an ED by two senior psychiatrists together, based on the diagnostic criteria of DSM‐IV‐TR, and then these patients were divided into the AN group, BN group, and other unspecified eating disorder group.
2. *Physical assessment*: To calculate BMI (kg/m^2^), the participants' weight and height were evaluated by a trained research assistant using a stadiometer. All patients were weighed in light indoor clothing without shoes.
3. *General demographic questionnaire*: This questionnaire was developed by the researchers and included study ID, patient name, gender, age, occupation, marital status, education level, BMI, age of onset, total duration of illness, onset inducement, current diagnosis, behaviors causing weight loss, and so on.
4. *The* EDI‐2 (Garner, 1991): The EDI‐2 is reliable and validated 91‐items, multidimensional, self‐report questionnaire that is designed to assess different cognitive and behavioral characteristics of EDs (Garner, 1991). It includes the 64 items (grouped into eight subscales: drive for thinness, bulimia, body dissatisfaction, ineffectiveness, perfectionism, interpersonal distrust, and interoceptive awareness, maturity fears) of the EDI‐I and adds 27 new items into three additional scales: asceticism, impulse regulation, and social insecurity. All of these items are answered on a six‐point scale: always, usually, often, sometimes, occasionally, and never, with values of 3, 3, 3, 2, 1, and 0, respectively. The higher the total score is, the more likely one is to have an ED. Chinese scholars have introduced and translated the EDI‐1 scale and conducted credibility and validity studies with good results among young Chinese females in Hong Kong and patients with AN in Beijing (Chen, Leung, Wang, & Tang, 2005; Zhang & Kong, 2004). As a result, the first eight subscales of EDI‐2 were used in data analysis.

### 2.3 Analytical methods

The latent structure of the sample was investigated by three taxometric procedures: Latent‐Mode Factor Analysis (L‐Mode), Mean Above Minus Below A Cut (MAMBAC), and MAXimum EIGenvalue (MAXEIG). The taxometric analyses were programmed using R2.12.0, and the algorithms for the taxometric procedures were obtained from Ruscio (2010).

#### 2.3.1 MAXimum EIGenvalue

The MAXEIG procedure (Waller & Meehl, 1998) specifies an indicator as an input variable and sets a certain number of cut‐points to form intervals (windows). The windows are allowed to overlap, and the overlapping ratio is variable. In the current MAXEIG analyses, we used 100 windows with 90% overlap. The covariance matrix of the remaining indicators within each window was calculated, and the largest eigenvalue was extracted. The extracted max eigenvalues were then plotted against the input variable. If the structure is taxonic, the plots tend to present a distinctive peak. On the contrary, dimensional data tend to produce relatively flat plots.

#### 2.3.2 L‐mode

L‐Mode (Waller & Meehl, 1998) is used to process the analysis of three or more indicators. It uses the Bartlett (19371937) method of factor score estimation to calculate a single latent factor and then plot the calculated case estimated scores. With enough indicators and validity, a dimensional structure would present a unimodal distribution, while a taxonic structure would present a bimodal distribution.

#### 2.3.3 Mean above minus below a cut

In the MAMBAC (Meehl & Yonce, 1994) analysis, two indicators are used: one for the input variable and the other for the output variable. When there are multiple variables, the remaining variables, other than the output variable, can be added as an input variable, or several variables are chosen to be added as an input variable; the other metrics are added as output variables. Then, according to certain rules, a cut‐point is determined for the input variables. Data higher than the cut‐point are classified into high‐score groups, while the others are classified into low‐score groups, and the differences between the means of these two groups are compared. The plots are formed with the input variable as the lateral axis and the difference between the two means as the vertical axis. A taxonic MAMBAC plot has a distinct peak, while dimensional plots result in a “U” shape graph.

#### 2.3.4 Comparison Curve Fit Indice

A comparison was made based on the relative fit of the research data plots to the simulated taxonic and dimensional curves and was assessed visually and with the objective comparison curve fit index (CCFI; Ruscio, Ruscio, & Meron, 2007). The CCFI is used to objectively determine the attributes of potential structures. The index ranges from 0 to 1, with 0.5 as the dividing line. If the index is closer to 0, a dimensional structure is more likely. If the index is closer to 1, a categorical structure is more likely. An index value from 0.45 to 0.55 is the fuzzy range of the index (Ruscio, Borkovec, & Ruscio, 2001), meaning the data distribution needs to be carefully explained if the index falls into this interval. CCFI indicators are well validated and have also been confirmed by many studies (Ruscio, 2007; Ruscio & Kaczetow, 2009; Ruscio & Marcus, 2007; Ruscio & Walters, 2009).

We used SPSS 20.0 (SPSS Inc., Chicago, IL) to analyze the demographic data of ED patients. Among the measurement data, the age at treatment and the age of onset were not normally distributed; therefore, they are described with medians (minimum, maximum). BMI conformed with a normal distribution and is, therefore, described by the mean value and *SD*.

## 3 RESULTS

### 3.1 Indicator validity

The following variables have been chosen to measure the severity of ED: z‐score of BMI, eight subscales (drive for thinness, bulimia, body dissatisfaction, ineffectiveness, perfectionism, interpersonal distrust, and interoceptive awareness, maturity fears) of the EDI‐2. This study used the score of each aspect as an indicator and analyzed the suitability of them for the further analysis in taxometric procedures. The within class correlations or nuisance covariances were measured. The selected indicators should sufficiently correlate within the full sample, at the same time having minimal correlations within the putative taxon and complement groups. As shown in Table 2, the results of preliminary analysis revealed that the nine EDs indicators demonstrated sufficient convergent validity and the scores of nuisance covariances were below the recommended threshold of 0.30 (Meehl, 1995). The indicators exceeded minimum validity criteria (Cohen's *d* > 1.25 *SD*; Ruscio et al., 2010) were selected, indicating adequate distinction between the putative taxon and complement group (Meehl, 1995; Beauchaine & Beauchaine, 20022002). There is only one validated indicator for the anorexia nervosa restricting type plus nonclinical comparison group, so this group did not enter subsequent taximetrics analysis. As shown in Tables 3 and 4, the indicators selected in anorexia nervosa binge‐eating/purging type plus nonclinical comparison group (AN‐BP group) and NN plus nonclinical comparison group (BN group) exceeded the recommended threshold for meaningful group differences. With large enough sample size, sufficient base rates, adequate nuisance covariances and validities, the data and indicators selected in this study were suitable for TA.

**Table 3:** Descriptive data for different indicators included in the taxometrics analysis of anorexia nervosa binge‐eating/purging type plus nonclinical comparison group

Indicator	Mean	*SD*	Range	Skew	Cohen's *d*
Indicator 1	1.66	3.78	0–21	3.19	2.51
Indicator 2	4.70	5.15	0–30	1.66	1.57
Indicator 3	3.74	4.77	0–30	2.63	1.56
Indicator 4	−0.33	1.11	10.85–26.62	−0.46	−2.07

*Note*. Cohen's *d* is measured in effect size units and represents the ability of each indicator to separate the putative taxon (i.e., AN‐BP) group from the putative complement (non AN) group. Indicator 1: bulimia; Indicator 2: ineffectiveness; Indicator 3: interoceptive awareness; Indicator 4: z‐score of BMI.

**Table 4:** Descriptive data for different indicators included in the taxometrics analysis of bulimia nervosa plus nonclinical comparison group

Indicator	Mean	*SD*	Range	Skew	Cohen's *d*
Indicator 1	5.08	5.19	0–21	1.09	1.63
Indicator 2	2.06	4.29	0–21	2.64	3.68
Indicator 3	7.94	5.45	0–27	0.75	1.25
Indicator 4	4.82	5.14	0–30	1.53	1.67
Indicator 5	3.93	4.81	0–30	1.87	1.82

*Note*. Cohen's *d* is measured in effect size units and represents the ability of each indicator to separate the putative taxon (i.e., AN‐BP) group from the putative complement (non AN) group. Indicator 1: drive for thinness; Indicator 2: bulimia; Indicator 3: body dissatisfaction; Indicator 4: ineffectiveness; Indicator 5: interoceptive awareness.

### 3.2 Anorexia nervosa binge‐eating/purging type plus nonclinical comparison group

As shown in Table 3, four indicators representing the bulimia, ineffectiveness, and interoceptive awareness subscales of the EDI‐2 and BMI were selected based on former evaluation process.

**Figure 1:**
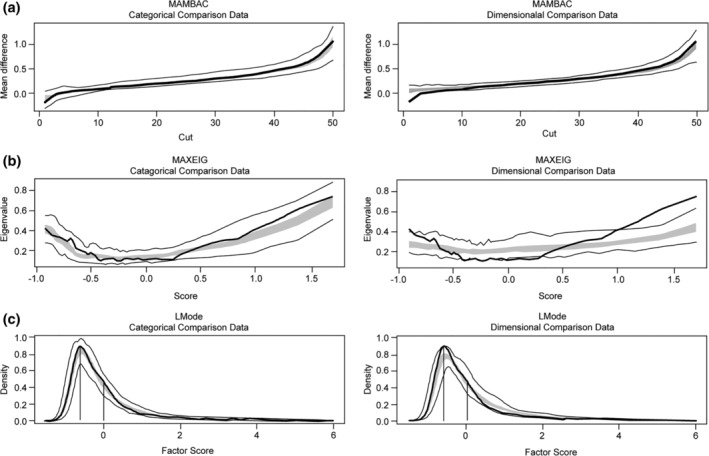
Taxometric analyses of anorexia nervosa binge‐eating/purging type and nonclinical comparison groups across panels (a)–(c). Mean CCFI = 0.702; final interpretation is categorical. *Note*. CCFI: comparison curve fit index; MAMBAC: mean above minus below a cut; MAXEIG: maximum eigenvalue; L‐mode: latent mode

In the MAMBAC analysis, we used 50 evenly spaced cuts beginning 25 cases from either extreme. An averaged curve was produced from the 12 separate curves. Comparing the averaged MAXEIG data curve with simulated taxonic and dimensional comparison plots, the average curve resembled the taxonic data. (Figure 1a). Moreover, the CCFI (0.694) indicated moderate support for a dimensional structure (Table 5).

**Table 5:** Base rate and comparison curve fit indice

	AN‐BP		BN			
	MAMBAC	MAXEIG	L‐Mode	MAMBAC	MAXEIG	L‐Mode
BR	0.829	0.878	0.269–1	0.168	0.119	0.259–1
CCFI	0.694	0.709	0.704	0.727	0.67	0.62
Average CCFI	0.702			0.672		

*Note*. CCFI <0.40 indicates a dimensional latent structure and CCFI >0.60 indicates a taxonic latent structure. Average CCFI: mean of the CCFIs in the three procedures; BR: taxon base rate; CCFI: comparison curve fit index.

In the MAXEIG analysis, we used 50 windows with a 90% overlap. As shown in Figure 1b, comparing the averaged MAXEIG data curve with simulated taxonic and dimensional comparison plots, the average curve resembled the taxonic data. The CCFI score was 0.709, which was above 0.6, supporting the categorical latent structures.

The results of the L‐mode analysis were consistent with those in MAMBAC and MAXEIG. As displayed in Figure 1c, the L‐MODE data curve was more consistent with the simulated categorical comparison plots. The CCFI was 0.704, which was higher than 0.6 and supported the taxonical latent structure.

Considering the results of all three taxometric procedures, each procedure provided convergent evidence favoring a taxonical latent structure of AN‐BP and nonclinical population.

### 3.3 Bulimia nervosa plus nonclinical comparison group

**Figure 2:**
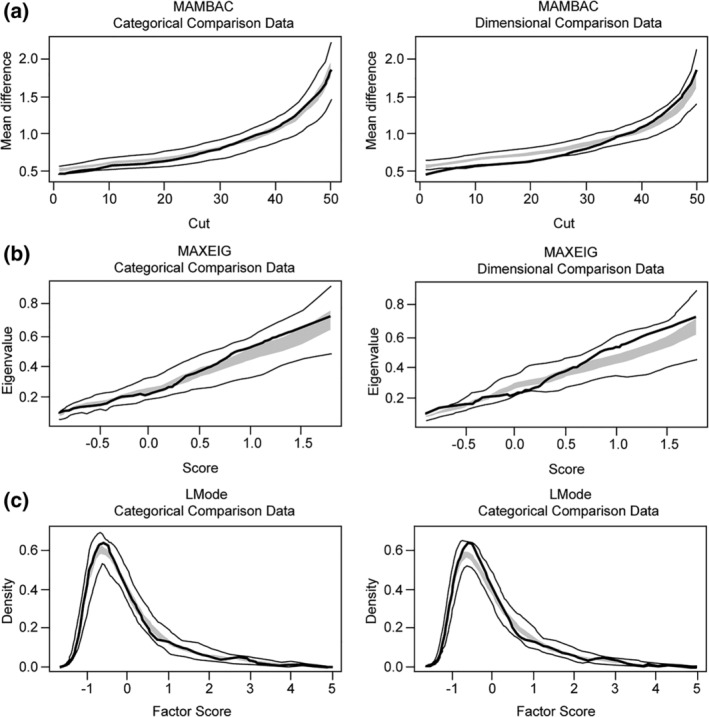
Taxometric analyses of bulimia nervosa and nonclinical comparison groups across panels (a)–(c). Mean CCFI = 0.672; final interpretation is categorical. *Note*. CCFI: comparison curve fit index; MAMBAC: mean above minus below a cut; MAXEIG: maximum eigenvalue; L‐mode: latent mode

Five indicators (drive for thinness, bulimia, body dissatisfaction, ineffectiveness, and interoceptive awareness) met validity criteria and were submitted to TA. All of the averaged data curves were highly consistent with simulated categorical curves (Figure 2). CCFI scores of MAXEIG (0.67), MAMBAC (0.727), and L‐Mode (0.62) have provided further support for a categorical solution. Thus, the present results provide convergent evidence to support BN existing as discrete typologies in Chinese sample.

## 4 DISCUSSION

Eating disorders were originally considered a product of western culture (Keel & Klump, 2003), and few cases in China were reported in the past. However, in the past three decades, with the economic development and impact of western culture, the incidence of EDs in China has shown an increasing trend. Although there is no nationwide epidemiological survey and regional epidemiological findings are rare (Qian et al., 2013), the number of ED inpatients in the Shanghai Mental Health Center for the recent 10 years (2004–2013) has increased threefold compared to the previous decade (1994–2003), and the average annual number of new cases of outpatients with EDs in the recent 5 years (2009–2013) is twice that of the previous 5 years (Chen, 2013). A survey of female college students in Shanghai showed that up to 17% of them display problematic eating behaviors according to the results of EDE‐Q questionnaires (Shi, Liang, & Li, 2009).

The present study is the first to classify ED patients by measuring weight level with BMI‐for‐age among the adolescents. It is also the first study using taxometric procedures to analyze the data in ED patients in mainland China.

Three most commonly used taxometric procedures (MAXEIG, L‐Mode, and MAMBAC) were applied and the results (0.694, 0.709, 0.704 in the AN‐BP group and 0.727, 0.67, 0.62 in the BN group, respectively) converge on a categorical distinction between specific ED subtype (AN‐BP and BN) with normalcy. The results of visual comparison also supported this conclusion.

Compared with previous studies, the results of this study supported the results of Williamson et al. (2002) and Gleaves et al. (2000), which reveals a taxonical latent structure of BN from those of persons with non‐pathological eating behaviors. Although the insufficient of sample size did not allow the comparison between each ED subtype, the results of AN‐BP group, which were proved to be taxonically distinct from nonclinical individuals, still supported that AN, binge‐eating/purging type might not fall on the same continuum with AN, restricting type (Gleaves et al., 2000), which as suggested to be continuous with normalcy in previous studies (Gleaves et al., 2000; Williamson et al., 2002).

The resulting Cohen's *d* value suggested the degree that indicators could influence the difference between the ED subtypes and nonclinical group, which could be considered as additional aspects to make evaluation in clinical practice. According to Cohen's *d* value, AN‐BP was differentiated primarily by the scale of bulimia, following by z‐score of BMI, ineffectiveness, and interoceptive awareness; for BN, it was bulimia, interoceptive awareness, ineffectiveness, drive for thinness, and body dissatisfaction in sequence. The behavioral indicators were consistent with the diagnostic criteria in current classification such as DSM‐5 and ICD‐10. Although compared to behaviors, the psychological aspects were not as objective and could not be dichotomized, reducing their practicability in clinical diagnosing process, they could be used to evaluate the severity of this disease and provide evidence for targets of further treatments.

The consistency of the results with other studies in western culture (Gleaves et al., 2000; Gleaves, Lowe, Green, et al., 2000; Williamson et al., 2002) has provided extra evidence for the generality of the latent structure of specific subtypes of EDs (AN‐BP and BN). However, in this study, the AN‐R group did not meet the requirement of TA because it demarcated from normalcy with one single feature (z‐score of BMI; Cohen's *d* = 2.43). Factor mixture analyses have been applied to solve this problem, using the simultaneous modeling of both latent categories and latent dimensions (Keel, Brown, Holland, & Bodell, 2012), which might be an optional method for our further studies.

Compared with the previous study in Hong Kong, this study has focus on another aspect of the latent structure of ED symptoms. The study in Hong Kong has measured the distinction within ED patients, while this study would like to answer whether the ED subtypes are categorically different from normalcy. The results of the two studies, which share a common cultural background, could be complementary, indicating that the latent structure of ED symptoms in Chinese patients was consistent with other western countries.

These results have given empirical evidence to inform clinical diagnosis and intervention in the future (Ruscio, Haslam, & Ruscio, 2006). Taken together with other studies, the results suggest the existence of symptoms rather than using the severity to make a diagnosis, supporting the rational of using categorical classification such as DSM‐5 or ICD‐10 in clinical practice. They have also supported the opinion that genetic or physical factors, instead of environmental and personal factors should be discussed more in further studies of pathogenesis (Williamson et al., 2005). Meanwhile, Williamson et al. have suggested that EDs might both have dimensional and categorical features and conceptualized three dimensions in the model of EDs: binge‐eating as a taxon, fear of fatness‐compensatory behaviors, and extreme drive for thinness as dimensional features. The hybrid model could facilitate the understanding of EDs, considering the possibility of dimensionality in other aspects of these diseases.

This study still has some limitation to be mentioned. First, it is a cross‐sectional retrospective research study, which means the data collected only reflected the situation at the moment when the patients were admitted to the hospital. However, these conditions (e.g., BMI, drive for thinness, and binge‐eating) might change later on. Patients might start to have fat‐phobic opinions as they gain weight, or their diagnoses might change (e.g., the transformation between AN and BN), which could skew the results. Second, TA was limited when more than two latent categories existing in the sample or only one single feature discriminated between potential categories (Keel et al., 2011). Third, the results of this study might not be generalizable to the Chinese population as the samples were only collected in one mental health center in Shanghai. Meanwhile, Shanghai has been influenced by western culture for a long time. The city's residents' eating behaviors might also be affected by western customs. As the result of this fact, further research with larger sample sizes and multi‐region studies is needed. Fourth, data in this study were mostly collected by self‐rating questionnaires. There might be a risk of response bias. Finally, lacking objective indexes, such as biochemical variables and prognosis, the variables included were limited.

Overall, this study has examined the latent structure of EDs using EDI‐2 in a Chinese sample with taxometric techniques, demonstrating that the latent structures of AN‐BP and BN are categorical rather than dimensional. These findings add evidence to the generality of current ED classification in Asian sample. Future studies should consider using hybrid model in data analysis, larger sample size and more variables such as biochemical and longitudinal data are also required.

## CONFLICT OF INTEREST

The authors declare no potential conflict of interests.

## AUTHOR CONTRIBUTIONS

Dr. Yuchen Zheng designed and conducted this study, including data analysis and writing the article. Dr. Qing Kang was responsible for collecting and entering data. Dr. Wenhui Jiang, Qiang Liu, Han Chen, Qing Fan, and Zhen Wang were responsible for recruiting, diagnosing, and classifying the patients. Professor Zeping Xiao revised the study design and the article. Dr. Jue Chen recruited and provided patients and revised the article.
